# Mucormycosis co-infection in COVID-19 patients: An update

**DOI:** 10.1515/biol-2022-0085

**Published:** 2022-08-10

**Authors:** Abdullah S. Alkhamiss, Ahmed A. Ahmed, Zafar Rasheed, Ruqaih Alghsham, Ali Shariq, Thamir Alsaeed, Sami A. Althwab, Suliman Alsagaby, Abdullah S. M. Aljohani, Fahad A. Alhumaydhi, Sharifa K. Alduraibi, Alaa K. Alduraibi, Homaidan T. Alhomaidan, Khaled S. Allemailem, Raya A. Alharbi, Samar A. Alamro, Arwa M. Alqusayer, Sahim A. Alharbi, Thekra A. Alharby, Mona S. Almujaydil, Ayman M. Mousa, Sultan A. Alghaniam, Abdulrhman A. Alghunaim, Rana Alghamdi, Nelson Fernández, Waleed Al Abdulmonem

**Affiliations:** Department of Pathology, College of Medicine, Qassim University, Buraidah, Saudi Arabia; Research Center, College of Medicine, Qassim University, Buraidah, Saudi Arabia; Department of Medical Biochemistry, College of Medicine, Qassim University, Buraidah, Saudi Arabia; Departments of Microbiology, College of Medicine, Qassim University, Buraidah, Saudi Arabia; Department of Food Science and Human Nutrition, College of Agriculture and Veterinary Medicine, Qassim University, Buraidah, Saudi Arabia; Department of Medical Laboratories Sciences, College of Applied Medical Sciences, Majmaah University, Majmaah, Saudi Arabia; Department of Veterinary Medicine, College of Agricultural and Veterinary Medicine, Qassim University, Buraidah, Saudi Arabia; Department of Medical Laboratories, College of Applied Medical Sciences, Qassim University, Buraidah, Saudi Arabia; Department of Radiology, College of Medicine, Qassim University, Buraidah, Saudi Arabia; Department of Family and Community Medicine, College of Medicine, Qassim University, Buraidah, Saudi Arabia; Department of Basic Health Sciences, College of Applied Medical Sciences, Qassim University, Buraidah, Saudi Arabia; Department of Histology and Cell Biology, Faculty of Medicine, Benha University, Benha, Egypt; Department of Clinical Nutrition, Qassim Health Affairs, Ministry of Health, Buraidah, Saudi Arabia; Private Health Sector, Qassim Health, Jeddah, Saudi Arabia; Department of Chemistry, Science and Arts College, Rabigh Campus, King Abdulaziz University, Jeddah, Saudi Arabia; School of Life Sciences, University of Essex, Colchester, UK

**Keywords:** mucormycosis, immunocompromise, COVID-19, rhino-orbito-cerebral, pulmonary, diabetes: diabetes ketoacidosis, corticosteroids

## Abstract

Mucormycosis (MCM) is a rare fungal disorder that has recently been increased in parallel with novel COVID-19 infection. MCM with COVID-19 is extremely lethal, particularly in immunocompromised individuals. The collection of available scientific information helps in the management of this co-infection, but still, the main question on COVID-19, whether it is occasional, participatory, concurrent, or coincidental needs to be addressed. Several case reports of these co-infections have been explained as causal associations, but the direct contribution in immunocompromised individuals remains to be explored completely. This review aims to provide an update that serves as a guide for the diagnosis and treatment of MCM patients’ co-infection with COVID-19. The initial report has suggested that COVID-19 patients might be susceptible to developing invasive fungal infections by different species, including MCM as a co-infection. In spite of this, co-infection has been explored only in severe cases with common triangles: diabetes, diabetes ketoacidosis, and corticosteroids. Pathogenic mechanisms in the aggressiveness of MCM infection involves the reduction of phagocytic activity, attainable quantities of ferritin attributed with transferrin in diabetic ketoacidosis, and fungal heme oxygenase, which enhances iron absorption for its metabolism. Therefore, severe COVID-19 cases are associated with increased risk factors of invasive fungal co-infections. In addition, COVID-19 infection leads to reduction in cluster of differentiation, especially CD4+ and CD8+ T cell counts, which may be highly implicated in fungal co-infections. Thus, the progress in MCM management is dependent on a different strategy, including reduction or stopping of implicit predisposing factors, early intake of active antifungal drugs at appropriate doses, and complete elimination via surgical debridement of infected tissues.

## 1 Introduction

Mucormycosis (MCM) is described as a rare invasive fungal infection with high morbidity and mortality. Order Mucorales belong to the class Mucormycetes, subsequently to the subphylum Mucoromycotina [1]. It is associated with immunocompromised patients. However, several immunocompromised with various conditions including those with uncontrolled diabetes mellitus (DM), diabetic ketoacidosis, open wound following trauma, prolonged neutropenia, HIV or AIDS infection, iron overload or hemochromatosis, malignancies, corticosteroid therapy, organ transplant, and severe burns predispose this infection [2]. Although Mucorales belongs to nonpathologic fungus, the prevalence in immunocompetent individuals is a result of the existence of an intact immunity through neutrophils, which eliminate their spores [2,3]. Recently, severe COVID-19 infection was added as a co-factor that might cause a significant and sustained lymphopenia, leading to developing opportunistic infections, either bacterial or fungal. The first case was reported with COVID-19, which developed pulmonary cavitary lesions due to Mucorales fungi co-infection with harmful complications [3]. In fact, Mucorales fungi can invade blood vessels, which causes ischemic necrosis, and can potentially invades various systems such as the lung, central nervous system (CNS), nose, sinuses, skin, orbit, jaw bones, joints, heart, and kidney and can be classified into the following forms: pulmonary, rhino-cerebral, sino-nasal, cutaneous, oral mycosis, gastrointestinal, and disseminated [4]. This review article aimed to update the MCM information involving fungus classification, epidemiology, etiology, diagnostic tools, clinical settings, and treatment recommendations for invasive mycosis, which were found via the available original articles, original case reports, or published case series. Thus, this effort benefits to analyze and understand MCM via the available literature.

### 1.1 What is MCM?

**Figure 1:**
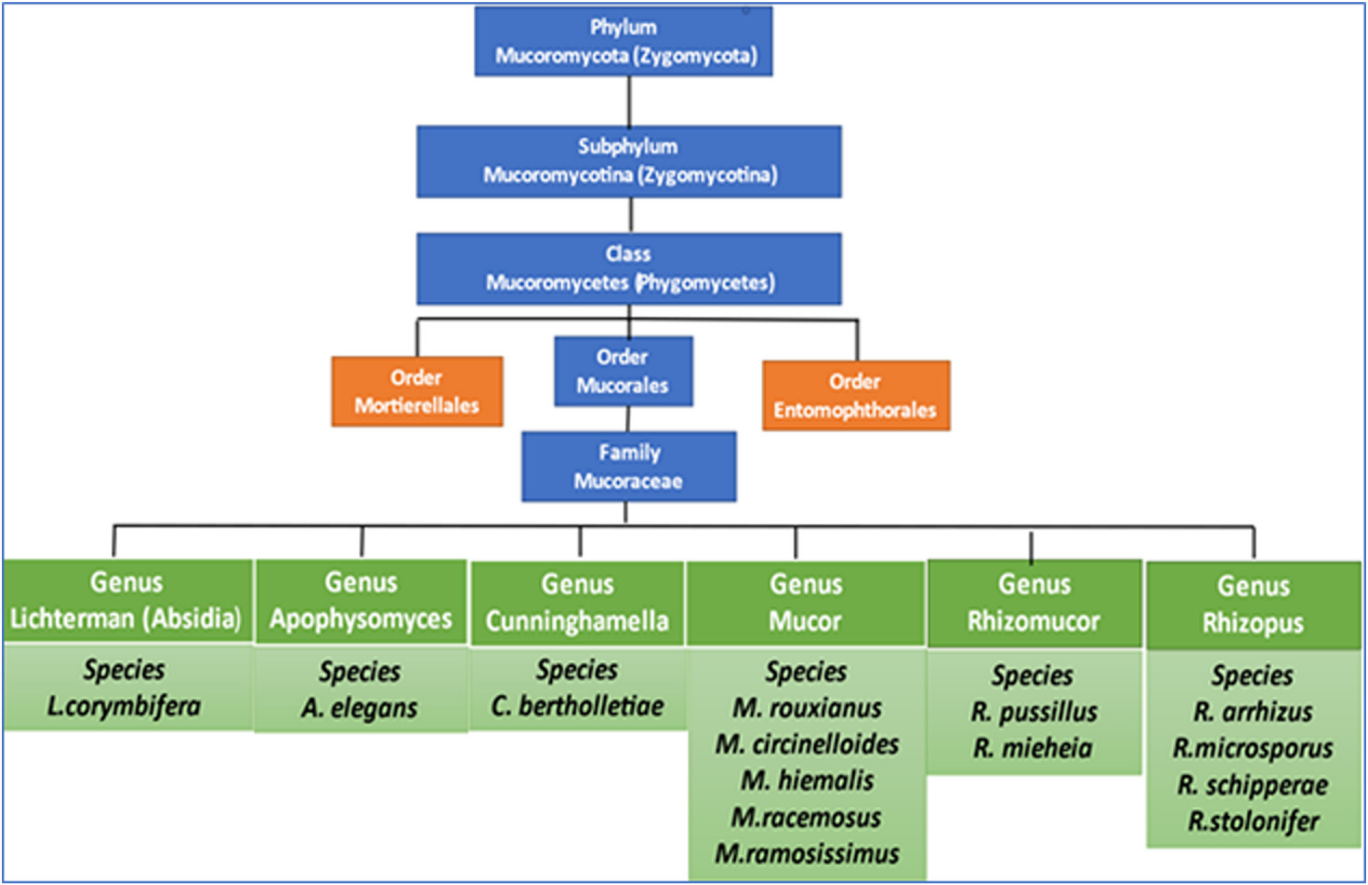
Classification of a common fungal species involved in the induction of mucormycosis.

MCM, previously known as zygomycosis, is the third most common invasive fungal infection after candidiasis and aspergillosis [2]. It is caused by mucoralean fungi (order Mucorales) [5]. Regarding a systematic position, the Mucorales order is considered more famous than the other zygosporic fungi orders (Entomophthorales and Mortierellales) [6]. Order Mucorales belonging to class Phycomycetes consequently to subphylum Mucoromycotina, thus phylum Mucoromycotina (Zygomycota) [7]. Mucorales containing the family Mucoraceae, which are classified into genus (species) *Lichterman* (formerly *Absidia*) (*L. corymbifera*), *Apophysomyces* (*A. elegans*), *Mucor* (*circinelloides*, *hiemalis*, *racemosus*, *ramosissimus*, *rouxianus*), *Rhizomucor* (*R. pusillus*, *mishear*), *Rhizopus* (*R. arrhizus*, *microsporus*, *schipperae*, *stolonifer*) (Figure 1) [8,9]. However, the order Entomophthorales contains two genera, Conidiobolus and Basidiobolus, which have been linked to human infections known as entomophthoromycosis. Mucorales are known to play a critical role in the development of MCM. In the last decade, according to molecular phylogenetic studies, the taxonomy of Mucorales has been modified widely and classified into 55 genera involving 261 species [10,11]. Thirty-eight species only have occasionally been implicated in MCM. But most of MCM infection (about 70–80%) is attributed to *Mucor rouxii*, *L. corymbifera*, *R. pussillus*, and *R. arrhizus* (formerly *R. oryzae*) [1,12,13,14] (Table 1).

**Table 1:** Update synonyms of medically important Mucorales species regarding to recent taxonomy [21]

Current nomenclature	Former names/synonyms
*Lichtheimia ramosa*	*Absidia ramosa, Mycocladus ramosus*
*Lichtheimia corymbifera*	*Absidia corymbifera, Mycocladus corymbifer*
*Lichtheimia ornata*	*Absidia ornata*
*Mucor circinelloides*	*Rhizomucor regularior, Rhizomucor variabilis*
*Mucor ardhlaengiktus*	*Mucor ellipsoideus, Mucor circinelloides*
*Mucor griseocyanus*	*Mucor circinelloides*
*Mucor janssenii*	*Mucor circinelloides*
*Mucor irregularis*	*Rhizomucor variabilis*
*Mucor lusitanicus*	*Mucor circinelloides*
*Rhizopus microsporus*	*Rhizopus azygosporus, Rhizopus chinensis, Rhizopus oligosporus, Rhizopus rhizopodiformis, Rhizopus tuberosus*
*Rhizopus arrhizus*	*Rhizopus oryzae*

### 1.2 Major epidemiological and clinical manifestations of MCM infection in patients with COVID-19

Currently, 213 cases have been confirmed as COVID-19–associated mucormycosis (CAM) in many countries like India (129 cases, 59.7%), followed by Egypt (44 cases, 20.37%), Turkey (12 cases, 5.6%), the United States (9 cases, 4.16%), Iran (4 cases, 1.8%), the Netherlands (4 cases, 1.8%), France (3 cases, 1.3%), Brazil (1 case, 0.4%), the UK (2 cases, 0.9%), Italy (1 case, 0.4%), Mexico (1 case, 0.4%), Spain (2 cases, 0.9%), and Austria (1 case, 0.4%). The data are presented in Table 2 and summarized in Table 3. The most common isolated Mucorales included *Rhizopus* spp., *Mucor* spp., *Lichtheimia*, and *Aspergillus* spp. Of note, most of them have rhino-orbito-cerebral MCM (100 cases, 45.3%), followed by rhino-orbital MCM (68 cases, 30.5%). Pulmonary MCM was observed in 24 cases (10.8%). However, sino-orbital was observed in 14 patients (6.3%), and gastrointestinal MCM was observed in 8 patients (4.0%). In addition, palatal ulcer MCM was observed in one case, abdominal MCM in two cases, musculoskeletal MCM in one case, maxillofacial MCM in two cases, and cutaneous MCM in one case. Regarding risk factors, the majority of patients with CAM have diabetes mellitus (93 cases, 42.7%), diabetes ketoacidosis (18 cases, 8%), hypertension (46 cases, 20.4%), or other comorbidities (8 cases, 3.6%). Patients who have received glucocorticoids were 67 cases (31.0%). A majority of patients were treated with amphotericin B as a primary antifungal agent with surgical debridement. Of these, 57 cases (27.3%) were deceased, 13 cases (6.0%) lost to follow-up, and 143 cases (66.6%) were alive. Data related to MCM and COVID-19 in the KSA and most Middle East countries are still limited.

**Table 2:** Summary of mucormycosis (MCM) co-infected with COVID-19

Number of cases	Country	Clinical presentation of MCM	Site of infection	Species	Underlying host risk factor	Diagnostics	Antifungal therapy	Outcome	Reference
1	USA	Rhino-orbital	Nasal sinus, orbit	NA	DKA, HTN, asthma	CT scan, histopathological and microbiological	Liposomal Amphotericin B	Improved	[94]
2	USA	Rhino-orbital-cerebral	Nasal sinus, orbit, CNS	NA	DM, DKA, received corticosteroids	CT scan, histopathological, and microbiological	Not mentioned	Died (*n* = 1), unchanged (*n* = 1)	[93]
1	UK	Pulmonary	Lung	NA	HTN, obesity, hypothyroidism	Q PCR, histochemical and immunohistochemistry examination	Not mentioned	Autopsy report	[109]
1	USA	Pulmonary	Lung	*Rhizopus* species	Not mentioned	CT scan, cultural examination	Amphotericin B	Died	[95]
11	India	Rhino-orbital-cerebral	Nasal sinus, orbit CNS majority	NA	DM, HTN	Cultural and histopathological examination CT scan	Amphotericin B	Died (*n* = 2), LFU (*n* = 5), improved (*n* = 4)	[106]
1	USA	Rhino-orbital	Nasal sinus, orbit	NA	DM, DKA	Histopathological and microbiological examination	Echinocandins and amphotericin B	Improved	[97]
10	India	Rhino-orbital-cerebral	Nasal sinus, orbit 1 = CNS	*Rhizopus* spp.	DM, DKT, received corticosteroid	Radiological and microbiological examination	Liposomal amphotericin B, dexamethasone	Died (*n* = 4), improved (*n* = 2), survived (*n* = 4)	[104]
1	USA	Pulmonary	Lung	*Rhizopus azygosporus*	Non mentioned	CT scan, histopathological Microbiological examination	Echinocandins and amphotericin B	Died	[99]
1	USA	Cutaneous	Skin	*Rhizopus microsporus*	DM, heart transplantation, received corticosteroid	CT scan cultural and microbiological examination	Amphotericin B, Caspofungin	Died	[100]
23	India	Rhino-orbital-cerebral	All-nasal sinus 10 = orbit 2 = CNS	NA	DM HTN received corticosteroid	MRI, CT scan	Surgical debridement, amphotericin B	LFU (*n* = 2), survived (*n* = 21)	[108]
1	Italy	Pulmonary	Nasal sinus lung	*Rhizopus* spp.	HTN	CT scan examination and histopathological examination	Amphotericin B	Died	[3]
1	France	Pulmonary	Lung	*Rhizopus microsporus*	Lymphoma, hematopoietic stem cell transplantation	Fungal qPCR CT scan	Liposomal amphotericin B	Died	[114]
1	India	Sino-orbital	Nasal sinus, orbit	*Rhizopus oryzae*	Received corticosteroid	MRI, histopathological examination	Surgery debridement, fluconazole, amphotericin B	Improved	[112]
2	Iran	Rhino-orbito-cerebral	2 = nasal sinus 2 = orbit 1 = CNS	NA	1 = DM, received corticosteroid	Histopathology CT scan	Endoscopic sinus debridement, amphotericin B	Died (*n* = 1), improved (*n* = 1)	[116]
6	India	Rhino-orbital-cerebral	All = nasal sinus, All = orbit 5 = CNS	NA	DM, received corticosteroid	Cultural, and histopathological examination, MRI	liposomal amphotericin B, posaconazole, surgical debridement	Improved	[103]
1	Mexico	Rhino-orbital	Nasal sinus, orbit	NA	DKA	Cultural examination, CT scan	Surgery debridement	Died	[118]
1	Austria	Pulmonary	Lung	*Rhizopus microsporus*	Leukemia	CT scan examination and histopathological examination	Voriconazole	Died	[119]
1	India	Rhino-orbital	Nasal sinus, orbit	NA	Not mentioned	MRI CT scan	Vancomycin, amphotericin B	Died	[110]
1	India	Pulmonary	Lung	*Rhizopus microsporus*	DM, HTN	CT scan, microbiological examination	Amphotericin B	Improved	[109]
1	Iran	Rhino-orbital	Nasal sinus, orbit	NA	NOD, received corticosteroid	Histopathological examination, CT scan	Unknown	Improved	[115]
1	India	Rhino-sinuses	Nasal sinus, orbit	NA	DM,	MRI, histopathological examination	Endoscopic sinus surgery, amphotericin B	Improved	[113]
1	India	Rhino-orbital–cerebral	Nasal sinus, orbit, CNS	NA	DM, NDD	CT scan, MRI, histopathological examination	Amphotericin B	Improved	[102]
1	USA	Pulmonary	Lung	*Rhizopus arrhizus*	DM, received corticosteroid	CT scan, histopathological, and microbiological examination	Not mentioned	Improved	[98]
10	India	Rhino-orbital	All = nasal sinus 2 = orbit 1- bone	NA	DM	Histopathological examination, CT scan, and MRI	Amphotericin B	Died (*n* = 1), survived (*n* = 3), improved (*n* = 5), LFU (*n* = 1)	[105]
11	India	Rhino-orbital-cerebral	Nasal sinus, orbit, CNS majority	NA	DM, HTN	Cultural and histopathological examination, CT scan	Amphotericin B	Died (*n* = 2), LFU (*n* = 5), improved (*n* = 4)	[106]
1	USA	Rhino-orbital -cerebral	Nasal sinus, CNS	*Rhizopus* species	DM, asthma, HTN, hyperlipidemia	CT scan, histopathological and microbiological examination	Endoscopic surgical debridement, vancomycin, cefepime, liposomal amphotericin B	Died	[96]
1	Brazil	Gastrointestinal	Gastrointestinal	NA	HTN	Esophagogastroduod, CT scan, histopathological examination	Unknown	Died	[101]
1	Turkey	Rhino-orbito-cerebral	Nasal sinus, orbit CNS	NA	DKA. received corticosteroid	Cultural examination, CT scan	Surgery debridement, amphotericin B	Died	[117]
1	Brazil	Palatal ulcer	Ulcerated, lesion with, coagulative, necrosis, hemorrhage, and abundant neutrophils	NA	DM	Histopathological examination, cultural examination, CT scan	Surgery debridement, amphotericin B	Survived	[117]
1	India	Rhino‑orbital	Periorbital pain followed by sudden onset of vision loss in the left eye	NA	DM	Histopathologic identification, cultural examination, CT scan	Surgery debridement, amphotericin B	Survived	[118]
31	India	Rhino‑orbital	Orbital cellulitis, ophthalmoplegia	NA	DM received corticosteroid (*n* = 19)	Cultural examination, CT scan	Surgery debridement, amphotericin B	Survived (*n* = 28), died (*n* = 3)	[119]
1	Iran	Rhino-orbital	Variable	NA	DM received corticosteroid	Histopathological examination	Combined antifungal, surgery debridement	Died	[120]
1	India	Gastrointestinal	Abdominal pain, nausea, vomiting	NA	NA	Histopathological examination, cultural examination, CT scan	Liposomal amphotericin B	Improved	[121]
10	India	Rhino-orbital	Headache and facial pain	NA	Received corticosteroid	Histopathological examination, cultural examination, CT scan	Amphotericin B, deoxycholate, and isavuconazole	Died (*n* = 1), survived (*n* = 9)	[122]
6	India	Paranasal sinusitis	Headache and facial pain	NA	DM	Histopathological examination, cultural examination, CT scan	Surgery debridement, amphotericin B	Improved (*n* = 6)	[123]
1	India	Abdominal	Abdominal pain, nausea, vomiting	NA	DM	Radiographic and histopathology in selected patients	Surgery debridement, amphotericin B	Died	[124]
1	India	Rhino-orbital	Acute loss of vision	NA	NA	Histopathological examination	Surgery debridement, amphotericin B	Recovered	[125]
2	Spain	Abdominal (*n* = 1), musculoskeletal (*n* = 2)	Abdominal and facial pain,	NA	DM, kidney transplantation (*n* = 1), HTN, steroid taken (*n* = 2)	Culture from the necrotic tissue	Surgery debridement, amphotericin B (*n* = 2), and initially isavuconazole, and subsequently posaconazole (*n* = 1)	Died (*n* = 1), survived (*n* = 1)	[126]
11	Turkey	Rhino-orbital (*n* = 8). Rhino-orbito –cerebral (*n* = 3)	8 = nasal sinus, 8 = orbit 3 = CNS	NA	DM (*n* = 8), HTN (*n* = 7), renal failure (*n* = 5)	Cultural examination, CT scan	Radical debridement, amphotericin B	Died (*n* = 7), survived (*n* = 4)	[127]
2	India	Maxillo-facial	Facial pain	NA	DM (*n* = 1), HTN (*n* = 2)	Maxillary biopsy, cultural examination, CT scan	Radical debridement, amphotericin B	Survived (*n* = 2)	[128]
8	Egypt	Pulmonary	Respiratory system, orbital cavities, ethmoidal and maxillary sinuses, nasal cavity, nasopharynx, carotid artery, hard palate, skin	Aspergillosis	DM (*n* = 6), chronic kidney disease (*n* = 2), hyperlipidemia (*n* = 2), HTN (*n* = 2), ischemic heart disease (*n* = 1), cerebral infarction (*n* = 1)	Radiographic and histopathology in selected patients	Amphotericin B, ambisome, itraconazole, surgical debridement, orbital enucleation, mechanical ventilation	Survived (*n* = 5), died (*n* = 3)	[129]
4	Netherlands	Pulmonary, rhino-orbital, rhino-sinuses	Respiratory failure. Acute-onset kidney failure, extensive sinusitis	*Rhizopus microsporus*, *Lichtheimia ramosa*, *A. fumigatus*, *R. arrhizus*	DM (*n* = 2), chronic lymphocytic lymphoma (*n* = 1), obesity (*n* = 1)	Radiographic and culture	Tocilizumab, dexamethasone, prednisone, amphotericin B, posaconazole, voriconazole, isavuconazole, surgical debridement, interferon-γ, mechanical ventilatio	Died (*n* = 3), survived (*n* = 1)	[130]
1	UK	Pulmonary, heart, hilar nodes, brain, pharynx, nasal mucosa, trachea	Acute anterior cerebral artery, pneumonitis	Aspergillosis	Hypothyroidism, steatohepatitis, thrombo-embolic disease	PCR, radiographic and culture	Mechanical ventilation, aspirin, LMW-heparin, hydroxychloroquine, azithromycin, meropenem, teicoplanin, argatroban, noradrenaline, vasopressin, gentamicin, tracheostomy, bronchoalveolar lavage	Died	[131]
36	Egypt	Rhino-orbital–cerebral (*n* = 29), sino-orbital (*n* = 7)	Facial painfacial numbness, ophthalmoplegia, and visual loss	*Mucor* and *Aspergillus* species	DM (*n* = 10), HTN (*n* = 6), leukemia (*n* = 1), pancreatic cancer (*n* = 1), CKD (*n* = 3), asthma (*n* = 3), cardiac (*n* = 1), hypothyroidism (*n* = 1), systemic lupus erythematosus (*n* = 2)	MRI, histopathological diagnosis	Amphotericin B, voriconazole, posaconazole, surgical debridement, mechanical ventilation	Died (*n* = 13), survived (*n* = 23)	[132]
2	France	Pulmonary		*Aspergillus*	Obesity (*n* = 2), kidney transplantation (*n* = 1), HTN (*n* = 1), dyslipidemia (*n* = 1)	MRI, histopathological diagnosis	One had no specific antifungal or COVID-19 treatments (died later). One received no specific COVID-19 therapies, but voriconazole, amphotericin B, caspofungin, and isavuconazole for fungal infections (alive)	Died (*n* = 1), survived (*n* = 1)	[133]

Q PCR, quantitive polymerase chain reaction; CT scan, computed tomography; MRI, magnetic resonance imaging; DM, diabetes mellitus; HTN, hypertension; CNS, central nervous system; GIT, gastro-intestinal tract; DKA, diabetic ketoacidosis; NOD, new-onset; NA, not available; LFU, lost to follow-up; LAMA, left against medical advice.

**Table 3:** Summary of mucormycosis infection in COVID-19 patients

Items	213 cases (%)
**Countries**	
India	129 (59.7)
Egypt	44 (20.37)
Turkey	12 (5.6)
USA	9 (4.16)
Iran	4 (1.8)
Netherland	4 (1.8)
France	3 (1.3)
Brazil	1 (0.4)
UK	2 (0.9)
Italy	1 (0.4)
Mexico	1 (0.4)
Spain	2 (0.9)
Austria	1 (0.4)
**Clinical manifestations**	
Rhino-orbito-cerebral	100 (45.3)
Rhino-orbital	68 (30.5)
Pulmonary	24 (10.8)
Sino-orbital	14 (6.3)
Gastrointestinal	8 (4.0)
Others	7 (3.1)
**Risk factors**	
Diabetes miletus	93 (42.7)
Diabetes ketoacidosis	18 (8)
Hypertension	96 (42.7)
Another comorbidity	8 (3.6)
Glucocorticoid’s intake	67 (31.0)
**Amphotericin B**	Major
**Surgical debridement**	Major
*Outcomes*	
Deceased	57 (27.3)
Alive	143 (66.6)
Lost follow-up	13 (6.0)

### 1.3 Etiology

When healthy individuals with immunocompetent inhale or ingest the fungal spores or lacerations of the mucosa via the nasal passage or oral cavity, they will not be implicated in immediate or potential harm as the phagocytic response will limit its prevalence. In contrast to the individual with immunocompromised and low polymorphonuclear leukocytes, the fungi become pathogenic when individuals invade by the fungal spores through lacerations of the mucosa in the oral or nasal cavity, may develop hyphae, and reach the paranasal sinuses. Subsequently, the disease can develop and diffuse to the cavernous sinus, the orbits, and the brain. It can also invade the arterial lamina and lead to tissue necrosis or thromboembolisms and infarctions of involved tissues. Then, the patient might suffer from orbital cellulitis, orbital apex syndrome, cerebritis or brain abscess, and a high mortality rate [15]. Mucorales and Entomophthorales orders are the most common etiological agents of MCM in humans. Several co-factors have been reported for a steady increase in MCM risk, such as:

1. Immunosuppression following solid organ, stem cells, and bone marrow transplants [16].
2. Severe neutropenia consequence of aggressive chemotherapy for hematological and solid malignancies since neutrophils are essential for phagocytosis of the fungus [17].
3. Rate of breakthrough invasive fungal infections is estimated between 4 and 10% of patients who are taking antifungal prophylaxis or treatment [18,19].

Uncontrolled DM and insufficient healthcare access can lead to an increase in MCM risk factors and hyperglycemia, which contributes to impaired neutrophil function and may cause MCM [20]. Therefore, DM is the most commonly known risk factor for MCM, especially during ketoacidosis. This is because ketones facilitate the fungi to utilize and produce ketoreductase, which facilitates its growth. In addition, hyperglycemia with ketoacidosis is also directly associated with the risk of MCM in different ways: (a) incidence of iron imprisonment because of hyperglycation of iron, which reduces the host protective system; (b) enabling tissue breakthrough by expressing the glucose-regulated protein 78 (GRP78) of cell receptor, which binds to Mucorales fungal species through the direct effect of hyperglycemia and indirectly by raising free iron levels; (c) deteriorating phagocytic functions and diminution the efficiency of chemotaxis; and (d) enhancing fungal existence via iron dissociation from breakthrough proteins [21]. GRP78 was involved within the cell receptor of endothelial vascular tissue [22].

### 1.4 Environmental factors and distribution of MCM

From an environmental perspective, these fungi are distributed in the soil, compost, animal feces, and decaying materials. In France, the analysis of collected soil samples from different geographical zones demonstrated that the most common species of Mucoralean fungi are *Mucor circinelloides*, *Rhizopus arrhizus* (synonym: *Rhizopus oryzae*), *Lichtheimia corymbifera*, *Rhizopus microsporus*, and *Cunninghamella bertholletiae* [8,9]. In comparison, a novel species have been discovered in Mexico (*Apophysomyces mexicanus*) [23]. Thus, the most common species of Mucorales found in the European countries are *Rhizopus* spp. (34%), *Mucor* spp. (19%), and *Lichtheimia* spp. (19%) [24], whereas in the United States, *Rhizopus* species were followed by *Mucor* species (19%), *Rhizomucor* species (7%), *Cunninghamella* species (9%), and *Lichtheimia* species (formerly *Absidia* species, 3%) [5]. Of note, MCM incidence among geographical regions is possibly due to the different natural habitats [24]. Conversely, the peak of MCM incidence has been investigated from September to November (autumn season) in some Middle East countries [8,25,26], while in European countries, most incidences are recorded in autumn and winter [27]. As well as in tropical and subtropical zones, the distribution of MCM was higher in autumn [28], suggesting that climate conditions may play a critical role in the prevalence of MCM and might have contributed to increasing airborne spore concentrations in autumn, whereas the fewer concentrations occur in summer. MCM incidence varies considerably from zone to zone regarding the susceptibility of co-factors. Several studies from Europe [24,29,30] reported that hematological malignancy was the most co-factor of infection, while in India [27,31,32], Iran [33], Mexico [31], Middle East, and North Africa [34], it was DM. Conversely, several studies have demonstrated that the underlying disease is associated with the site of infection [13,28,35]. However, hematological malignancies and neutropenia are correlated with pulmonary MCM, whereas DM is correlated with sinusitis and rhino-cerebral disease, while trauma usually leads to cutaneous MCM. DM and ketoacidosis DM is contributing to underlying disease in cases with MCM globally [13,35].

### 1.5 Diagnosis

Fast treatment of MCM disease is considered a big challenge. Therefore, the accuracy of diagnosis is very important for helping the treatment and reducing the high mortality (85%, concerned with late or incorrect diagnosis) [2]. Continuous development and discovering an accurate diagnosis are still essential because of the indirect clinical markers/symptoms for confirming MCM. Update of studies is required to understand the epidemiology and diagnosis of this disease among various regions worldwide. In spite of this, multiple studies reported the difficulty in establishing a rapid diagnosis [36]. The first step in the diagnosis begins with suspicion of the occurrence of MCM infection, especially the candidate diseases as co-factors. Hence, progress in diagnosis has affected the increasing reports on invasive MCM in susceptible patients like those with diabetic ketoacidosis secondary to uncontrolled DM, HMs, solid organ transplant, chronic respiratory diseases, and corticosteroid therapy [37]. Although, a conventional clinical path for diagnosis lacks specificity and sensitivity. It is still a basic requirement for the initial diagnosis of MCM when a high indicator of suspicion, via estimate of host factors and fast evaluation of clinical appearances. For example, the initial investigation of gastric MCM by endoscopic examination usually reveals an ulcer with necrosis, ultimately presenting an adherent, thick, green exudate [38]. Histopathology and culture are the fundamentals of diagnosis [13,39]. Prompt microscopy examination of clinical biopsy specimens is carried out, perfectly using optical brighteners such as Blankophor and Calcofluor [40,41,42]. White in clinical specimens gives a fast visualization of the characteristic fungal hyphae of MCM, in this case requiring a fluorescent microscope [2,43]. Also, in the case of diplopia in diabetic patients, pleuritic pain may be an indicator of this infection, and it is essential to obtain a specimen for traditional examination by histopathological and microbiological examinations or by advanced molecular techniques. The markers and symptoms that involve diplopia are cranial nerve palsy, proptosis, sinus pain, orbital apex syndrome, periorbital swelling, and ulcers of the palate [42]. In addition, radiologically, various (≥10) nodules and pleural emissions can reveal the association with pulmonary MCM [44]. Other pathways are computerized tomography (CT) scans with high-resolution and magnetic resonance imaging (MRI), which indicate the existence of MCM [2,13,45]. When studying the CT features of COVID-19–associated pulmonary mucormycosis situations, it was discovered that consolidation and cavitation were the most common computed tomography imaging findings (69%), probably because of delayed disease diagnosis [46]. According to another CT imaging study, almost all the patients (95.7%) displayed signs of pansinusitis. The infection spread beyond the paranasal sinuses was seen in 78.7% of cases, with orbital invasion 40.4% being the most prevalent [47]. On the contrary, MRI is useful for soft tissue imaging and determining the severity of disease [48].

Conversely, identifying different fungal species is important for the best epidemiological understanding of MCM and may be of significance for outbreak determinations. Mucorales fungi can be easily identified in culture. One study described the morphological features alone, and when distinguished by specialists in the fungal investigation, a high level of accuracy can be obtained. [49,50] However, identifying species is still difficult and may face failures in speciation, whereas immunohistochemistry using monoclonal antibodies against *R. arrhizus* can differentiate aspergillosis from MCM (100% high sensitivity and 100% high specificity for MCM) [51,52]. Recently, a molecular diagnosis is required either for the identification or detection of MCM [53]. It involves conventional polymerase chain reaction (PCR), [54,55,56], restriction fragment length polymorphism (RFLP) [56,57], and DNA sequencing [2,58]. Indeed, several PCR-based techniques have been developed, such as nested PCR, real-time PCR [59,60] (quantitative PCR (qPCR)), nested PCR combined with RFLP [61], PCR coupled with electrospray ionization mass spectrometry [62], and PCR/high-resolution melt analysis [63]. All assays mentioned earlier can be applied for either the identification or the detection of Mucorales. Most of the molecular assays are ribosomal targets (18S, 28S, and internal transcribed spacer) or other DNA targets (the high-affinity iron permease I gene FTRI or cytochrome b) [53,64,65,66,67]. Several studies have been performed by using either fresh tissue specimens or formalin-fixed paraffin-embedded [68,69,70], yet resulting in different performances. Recent tools directed at molecular assays from blood serum [68,71,72,73] have yielded promising clinical data with the advantage of early diagnosis in comparison with culture. Moreover, qPCR has been performed to identify circulating *Mucor*/*Rhizopus*, *Lichtheimia*, and *Rhizomucor* DNA in the serum [74]. However, some studies recommended that molecular diagnosis can be conducted in addition to conventional diagnosis tools [75].

### 1.6 COVID-19 and mucormycosis co-infections

Based on clinical manifestations, invasive MCM can be classified by the anatomic site affected into six main forms: pulmonary, rhino-orbital-cerebral, cutaneous, sinus, gastrointestinal, disseminated, and rare forms, such as endocarditis, osteomyelitis, peritonitis, and renal infection [76,77,78]. Any of Mucorales species can infect any of these sites. Fungal infection can invade these sites by the incorporation or implantation of the fungal spores through oral, nasal, and conjunctival mucosa (rhino-orbital-cerebral), by inhalation (pulmonary), or by the ingestion of contaminated food (gastrointestinal), as they rapidly colonize nutriments rich of glucose for its principal energy source [79]. Then the common ratio of invasive MCM was evaluated as follows: sinuses (39%), pulmonary (24%), and cutaneous (19%) [80]. Dissemination developed in 23% of this infection [81,82]. However, these values may be changeable depending on the incidence of another co-factor such as COVID-19. The first report has suggested that COVID-19 patients might be susceptible to developing invasive fungal infections by different species such as candidiasis, invasive aspergillosis, and pneumocystis jiroveci [83]. However, this report did not include MCM as a co-infection with the COVID-19 pandemic. Subsequently, the case report presented a patient with MCM without any conventional risk factor. The infection was confirmed after being diagnosed with COVID-19 and has been treated with broad-spectrum antibiotics and corticosteroids (which are critical factors for invasive fungal infection) [78,84]. Despite co-infection among patients with COVID-19 has been shown only in severe cases [82,85,86], superinfections in viral pneumonia remain unclear. The first reason is due to challenges confused by the conventional diagnosis of Mucorales. The second is due to the fact that many centers minimized direct testing of at-risk respiratory samples during the current COVID-19 pandemic to diminish exposure to the virus [87,88,89,90]. Subsequently, several reports confirmed increasing cases of MCM in different countries, particularly in India, after COVID-19. Of their findings of an even larger case series of MCM cases in COVID-19, about 80% of cases had DM and more than 75% of cases received a course of corticosteroids. Therefore, these findings indicated that the common triangle associated with COVID-19 and MCM co-infection are DM, diabetic ketoacidosis (DKA), and corticosteroids (Table 2) [3,89,90,91,92,93,94,95,96,97,98,99,100,101,102,103,104,105,106,107,108,109,110,111,112,113,114,115,116,117,118,119,120,121,122,123,124,125,126,127,128,129,130,131,132,133]. However, some finding in case of rhino-orbital MCM was associated with ketoacidosis DM and infection with severe acquired respiratory syndrome coronavirus 2 [134]. Song et al. have reported an algorithm for the early diagnosis and management of common invasive fungal infections such as MCM, aspergillosis, candidiasis, and cryptococcosis during the COVID-19 pandemic [135]. However, factors leading to increased incidence of opportunistic fungal infections during the COVID-19 pandemic may lead to immunosuppression due to high-dose corticosteroids in the management of the pandemic, thus leading to immunosuppression [136]. Corticosteroid intake during COVID-19 infection often leads to uncontrolled diabetes and precipitation of ketoacidosis. Thus, acidosis (low pH) is considered a favorite media for *Mucor* spores to grow. In addition, corticosteroid intake also reduces the phagocytic activity of white blood cell (WBC) and causes deterioration of bronchoalveolar macrophages migration, ingestion, and phagolysosome fusion, leading to exposure risk of diabetic people to MCM [89]. Consequently, a report described one case with SARS-CoV-2 infection who developed a cavitary pulmonary MCM [137]. Patients with severe acute respiratory syndrome coronavirus 2 (SARS-CoV-2) infection might develop coronavirus disease (COVID-19), which can be associated with significant and sustained lymphopenia compromising the immune system, especially in the most severe cases [3,138,139,140,141]. Recent findings described that a significant decrease in lymphocyte count and an increase in neutrophil count together with an inflammatory storm occur more frequently in patients who developed severe COVID-19 and co-infections [141]. Pathogenic mechanisms in aggressive MCM involve reduced phagocytic activity, attainable quantities of ferritin attributed to the displacement of protons by transferrin in diabetic ketoacidosis, and fungal heme oxygenase, which enhances iron absorption for its metabolism. Therefore, severe COVID-19 cases with hemato-lymphohistiocytosis syndrome are associated with the increasing risk factors of invasive fungal co-infections [90]. Clinically, cell counts exhibited that there was an increase in neutrophils and WBC count, but a decrease in lymphocytes progressively [3]. In addition, corticosteroid intake also reduces the phagocytic activity of WBC and causes deterioration of bronchoalveolar macrophages migration, ingestion, and phagolysosome fusion, leading to a diabetic people exposure risk to MCM. Conversely, the presence of free iron is an ideal co-factor for MCM. Hyperglycemia leads to glycosylation of transferrin and ferritin, and minimizing iron-binding leads to a raised ratio of free iron. Moreover, an increase in interleukin (IL)-6 among COVID-19 patients also causes an increase in free iron by elevating ferritin levels, and acidosis increases free iron by reducing the ability of transferrin to chelate iron [89]. In addition, COVID-19 infection drastically affects the immune system via hyperexpression of booth proinflammatory (IL-1, IL-2, IL-6, and tumor necrosis factor (TNF-α)) and anti-inflammatory (IL-4, IL-10, and IL-17) cytokines [4,139] and hypoexpression of interferon (IFN)-γ. With an increase in neutrophiles, impaired lymphocyte-mediated immunity (Th1 and Th2) occurs. Subsequently, COVID-19 infection reduces the cluster of differentiation, especially CD4+ and CD8+ T cell counts, which may be highly implicated in fungal co-infections [141]. Critical cases, especially those admitted to intensive care units and those requiring longer hospital stays might be more prospective to evolve fungal co-infections [142]. Therefore, it is to be noted that severe Covid-19 susceptible to opportunistic fungal co-infections, especially MCM.

### 1.7 Molecular mechanism of mucormycosis in COVID‑19 patients

**Figure 2:**
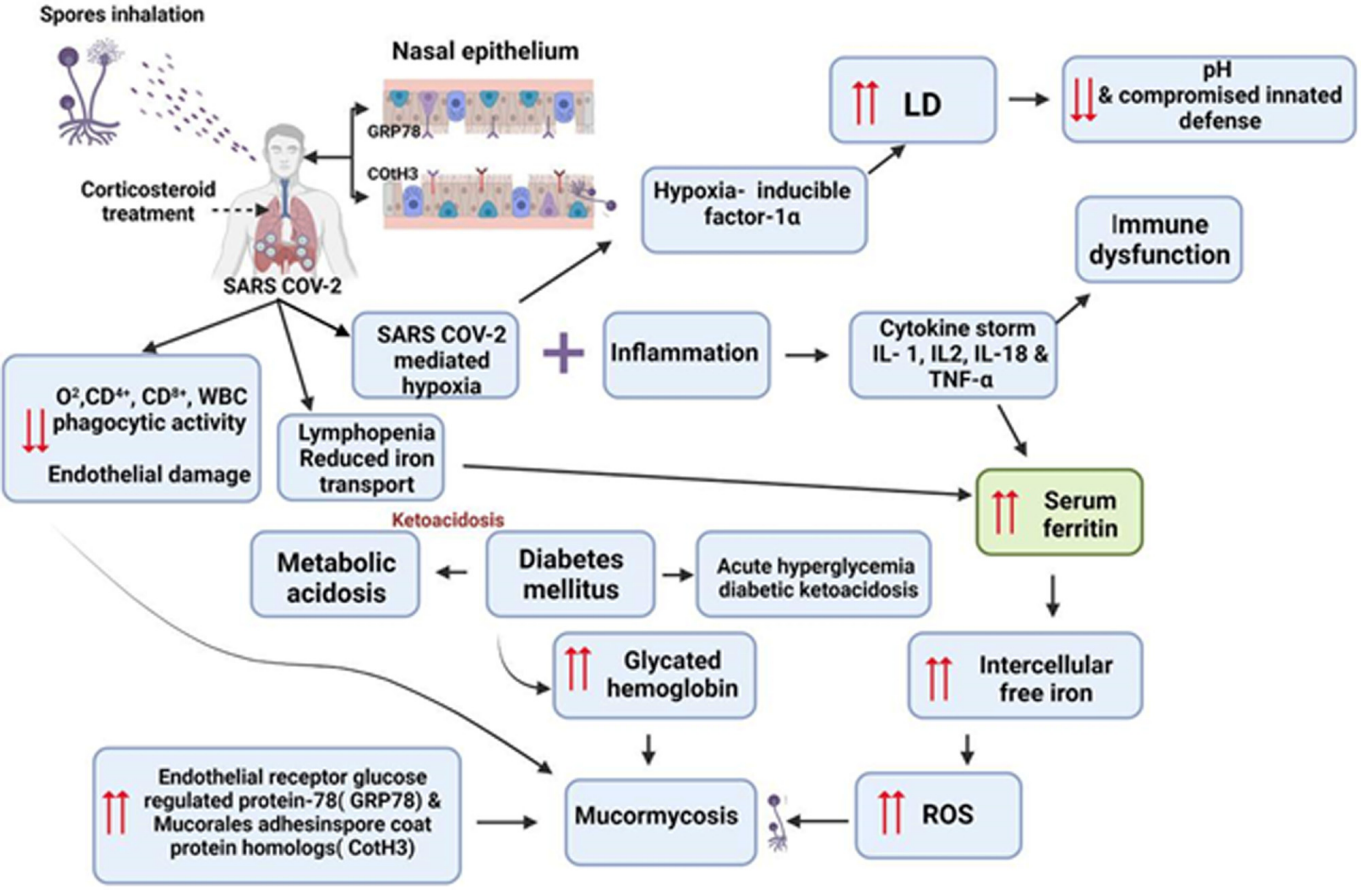
Proposed scheme revealed different pathways of mucormycosis post-COVID-19 infection. During fungal spore inhalation, fungal cells spore coat protein Cot H3 recognizes the GRP78 on nasal epithelial cells to invade, colonize, and lyse host cells. The overall potential such as corticosteroid therapy, immunocomprised, DM, DKA, hyperglycemia, hypoxia, cytokine storm, hyperferritinemia, neutropenia, metabolic dysfunction, LD, and ROS make COVID-19 infected patients increased growth mucormycosis. Abbreviations: GRP78, glucose-regulated protein 78; DM, diabetes mellitus; DKA, diabetic ketoacidosis; LD, lactate dehydrogenase; ROS, reactive oxygen; IL, interleukin.

The invasion of fungi usually starts by inhalation of spores during respiration (sometimes from ears), ingesting contaminated food, or invading through injuries/abrasion in the epidermis. Endothelialitis is the one factor of SARS-CoV-2 that might reveal the risk of MCM co-infection in COVID-19 patients. [143,144] Damage to endothelium promotes angio-invasion and prevalence of MCM. In addition, low pH, hyperglycemia, hyper-ferritin, and hyper-ketone due to COVID enhance an appropriate environmental condition for GRP78 of endothelial cells and fungal ligand spore coating homolog (CotH) protein. Molecular explanation between fungal CotH protein and nasal GRP78 enhances adherence in the early stage of infection (Figure 2). Then, the CotH surface proteins might act as invasion enzymes to cause the infection [145]. In the sequent step of infection, patients with the high levels of CotH and GRP78 lead to trapping of fungi inside sinus cavities (Figure 2) [146,147]. Then, fungal cells elongate into tube-like hyphal strands and disseminate into the sinuses, lungs, skin, soft tissues, and bloodstream, causing tissue and epithelium penetration (Figure 2). The second current pathway, corticosteroid intake, is the mainstay therapy in severe COVID-19 patients [148]. However, corticosteroid intake reduces the phagocytic capacity of WBCs, displaying patients’ fungal infections. In addition, new-onset corticosteroid-induced DM or exacerbated chronic DM with or without DKA might promote the replication of MCM. The COVID-19 itself launches a chain of events that might render patients prone to MCM infection. Thus, there are expected mechanisms through which SARS-CoV-2 infection might lead to MCM susceptibility. Dramatic elimination in the total number of T-cells involving both CD4+ and CD8+ groups in severe cases of COVID-19 causes significant lymphopenia successive an immunocompromised state and then susceptibility to MCM [4]. During severe COVID-19 infection, inflammatory cytokines such as IL-6, IL-2R, IL-10, and TNF-alpha elevate and lead to a term called “cytokine storm,” resulting in lymphopenia [4]. Consequently, cytokine storm raises iron levels and reduces its export. As a result, iron accumulates inside the cells. Iron overload causes tissue damage and necrosis. In addition, free iron is ideal for growth and spreading MCM (Figure 2) [149,150]. Moreover, SARS-induced pneumonia results in atelectasis, which results in COVID-19-contributed silent hypoxia. Usually, the hypoxia-inducible factor 1 (HIF-1) stays inactive in normal conditions. During hypoxic conditions of COVID-19, the HIF-1α transcription factor subunit has a risky role in the expression of the COVID-19 receptor gene (*ACE-2* gene), and hypoxia causes endothelium damage and upregulation of adaptive and innate immunocytes [151,152]. The overall effect of HIF-1 results in raised localized inflammation and tissue damage in COVID-19 patients. In addition, COVID-19 infection has also been demonstrated to impact ferritin metabolism. Therefore, several studies have reported hyperferritinemia syndrome post-COVID-19 infection. Thus, high free iron levels lead also to tissue damage via the production of reactive oxygen species (ROS) [150,153,154,155,156]. The overall potentials such as corticosteroid therapy, immunocompromised state, DM, DKA, hyperglycemia, hypoxia, cytokine storm, hyperferritinemia, neutropenia, metabolic dysfunction, and ROS make COVID-19–infected patient more susceptible to MCM. These main symptoms increased the body temperature, and altered osmolarity make an ideal environment for the growth and development of MCM (Figure 2).

### 1.8 Antifungal treatments

Progress in management and treatment of MCM is dependent on a different strategy, including reduction or stopping of implicit predisposing factors, early intake of active antifungal drugs at appropriate doses, complete elimination via surgical debridement of infected tissues, and administration of assistant therapies [57]. The therapy includes available antifungal medicines such as azithromycin, oseltamivir, ceftriaxone, amphotericin B (liposomal), cefepime, meropenem, linezolid, caspofungin, vancomycin, piperacillin, tazobactam, and oseltamivir. Intravenous amphotericin B (a lipid formulation) is the best and most common medicine for initial treatment, especially with COVID-19 co-infection. Despite surgical operations and available antifungal drugs, the prognosis for recovery from MCM is poor [95,106] (Table 2). Kieliszek and Lipinski [157] suggested that the sodium selenite, but not selenate, possibly oxidizes viral thiol groups in the virus protein disulfide isomerase, coating it incapable to penetrate the host cell membrane. In this way, the sodium selenite inhibits invasion of viruses into the healthy cells and diminishes their infectivity [157]. Thus, this simple chemical compound is potentially applied to the recently infected COVID-19 patients all over the globe.

## 2 Conclusion

The increase in MCM worldwide due to co-factors, especially diabetes with or without ketoacidosis, leads to outbreaks of corticosteroid intake during the incidence of COVID-19 (increases in blood glucose lead to opportunistic fungal infection). Outbreaks of COVID-19 are accompanied by immunocompromised through hyperexpression of both proinflammatory (IL-1, IL-2, IL-6, and TNF-α) and anti-inflammatory (IL-4, IL-10, and IL-17) cytokines, and vice versa with IFN-γ. With an increase in neutrophils, impaired lymphocyte-mediated immunity (Th1 and Th2) occurs. Subsequently, COVID-19 infection leads to a reduction in the cluster of differentiation, especially CD4+ and CD8+ T cell counts, which may be highly implicated in fungal co-infections. Therefore, it is to be noted that severe COVID-19 susceptible to opportunistic fungal co-infections, especially MCM. Every effort should be made to progress in the management and treatment of MCM is dependent on a different strategy, including reduction or stopping of implicit predisposing factors, early intake of active antifungal medicines. Corticosteroids were used only in cases co-infected with COVID-19 to reduce the load of mortal MCM.
